# A prospective study of the demographics, management and outcome of patients with acute kidney injury in Cape Town, South Africa

**DOI:** 10.1371/journal.pone.0177460

**Published:** 2017-06-01

**Authors:** Thandiwe A. L. Dlamini, Peter J. Heering, Tawanda Chivese, Brian Rayner

**Affiliations:** 1 Division of Nephrology and Hypertension, University of Cape Town, E13 Groote Schuur Hospital, Cape Town, South Africa; 2 Faculty of Medicine, Heinrich Heine University, Dusseldorf, Germany; 3 Chronic Disease Initiative for Africa, Department of Medicine, Faculty of Medicine and Health Sciences, University of Cape Town, South Africa; University of Sao Paulo Medical School, BRAZIL

## Abstract

**Aim:**

To study the demographics and outcome of acute kidney injury (AKI) at Groote Schuur Hospital, Cape Town, South Africa.

**Methods and findings:**

A prospective observational study of AKI fulfilling the Kidney Disease: Improving Global Outcomes definition, from 8 July 2012 to 8 July 2013. Ethics approval was granted by the University of Cape Town Human Research Ethics Committee. Consent was waived because patient data was de-identified and patient management was not adversely affected by the study. A clerking sheet was used for data collection. Patients were reassessed after 3 months. Main outcomes were renal recovery and 3 month mortality. Descriptive statistics and multivariate logistic regression were carried out for risk factors.

Over this period there were 10,750 hospital admissions and 366 patients with AKI giving an incidence of 3.4%. Median age was 44 years (IQR 14–82) and 214 (58.5%) were male, with 152 (41.5%) female. Most, 265 (72.4%), had community acquired AKI. Common underlying comorbidities were hypertension (n = 152, 41.5%), diabetes mellitus (n = 65, 17.8%) Human immunodeficiency virus (HIV) (n = 75, 20.6%), heart disease (n = 58, 16.1%), and chronic kidney disease (n = 37, 10.1%). Renal biopsies were performed in 36 (9.8%) patients. In total, 202 (55.2%) patients were in the intensive care unit, and of the whole study population 204 (55.7%) were dialysed. Those admitted to ICU who required dialysis amounted to 145 (39.6%). The overall 3 month mortality was 38.8%. Among the 145 patients dialysed in ICU, there were 71 deaths (49%) at 3 month follow up. Of the 119 patients with follow up serum creatinine, 95 (79.8%) had full renal recovery, and 4 (3.4%) had end-stage renal disease. On multivariate analysis, mechanical ventilation was associated with 3 month mortality (OR 2.46, p-value 0.019, 95% CI 1.41–4.03). Sepsis had a borderline significant association (OR 1.83, P-value 0.066, 95%CI 1.02–3.27), as did prolonged time to dialysis (OR 1.93, p-value 0.08, 095% CI 0.93–4.03). HIV status did not affect outcome. The main study limitations were the large numbers of patients with AKI stage 3, reflecting the fact that the institution is a tertiary referral centre and that patients with earlier stages of AKI tended not to be referred. Another study limitation was the low number of patients who were available for follow up for 3 month serum creatinine.

**Conclusions:**

The incidence of AKI in the population studied is 3.4% of hospital admissions and carries a high mortality risk, most significant in mechanically ventilated patients. Sepsis and late dialysis initiation may carry a risk of mortality, but HIV infection did not affect outcome. Follow up of patients at least 3 months after an episode of AKI is essential to detect and appropriately manage those with incomplete renal recovery. In this study 36 patients underwent a kidney biopsy, and in many of these the results guided patient management. This study demonstrates finally that it remains imperative that clinicians actively pursue underlying causes of acute decline in renal function, including urine analysis, renal ultrasonography and if indicated and safe, a renal biopsy.

## Introduction

Many studies have high-lighted the increased risks associated with AKI, in terms of in-hospital mortality, progression to end stage renal disease, accelerating progression of established chronic kidney disease (CKD), and increased cardiovascular risk [1,2]. Few data are available on AKI in African countries. In addition, access to renal replacement therapy is limited to fewer than 5% of patients who need it, especially in sub-Saharan Africa, resulting in death from a preventable cause [3]. The International Society of Nephrology has set a goal of zero by 25 –zero deaths from AKI by the year 2025 in low income countries [4]. AKI is therefore a condition that developing countries cannot afford to ignore, and appreciation of its local pattern can help inform policies on its prevention and management. Groote Schuur Hospital is a large academic institution with access to dialysis and serves the largely underprivileged population of Cape Town where there is a high prevalence of HIV infection.

Four important insights were gained from the study, and we believe that these can contribute to the existing body of knowledge on AKI.

Firstly, we found that the majority of patients with nosocomial AKI had concomitant sepsis (62.4%) and were referred with advanced AKI stage 3 (71.3%). This is valuable feedback to clinicians as it highlights the importance of optimal management of sepsis and alertness for deterioration in kidney function in these patients. Adoption of a simple tool such as an early warning score system can enable early detection and management of deteriorating patients and minimise the risk of AKI.

Secondly, we were able to highlight the importance of patient follow up after an episode of AKI. Of those followed up, 16.8% had only partial renal recovery and 3.4% had end stage renal disease. Those with partial renal recovery had the opportunity of being followed up and managed as per CKD guidelines to slow progression to end stage renal disease.

Thirdly, we were able to highlight the fact that acute deterioration in kidney function has a wide spectrum of potential causes that need to be identified if the patient is to be optimally managed. We found urinary tract obstruction in 4.3% of patients and 36 patients underwent renal biopsies. In many of these biopsied patients the diagnosis guided patient management. For example glomerulonephritis was detected in 52.8% of the 36 patients. This is particularly important in light of the current classification of AKI, where the pathophysiology of acute tubular necrosis is emphasized.

Lastly, we found that while HIV is an important co-morbidity to consider in South Africa, it was not a predictor of mortality in the patients that we studied.

The overall aim of the study was to gain insight into the demographics, pattern and outcome of patients with AKI, as well as to determine ways to minimise its occurrence. The study was able to fulfil most of these aims.

## Methods

We conducted a prospective cohort study of patients referred to Groote Schuur Hospital Renal Unit with AKI, from the 8th of July 2012 to the 8th of July 2013. Ethics approval was granted by the University of Cape Town Human Research Ethics Committee and consent was waived because patient data was de-identified and patient management was not adversely affected by the study. Patients were included if they were above the age of 13, with a native kidney. Patients with baseline serum creatinine greater than 354μmol/L were excluded. This value was chosen because it is the one used in the RIFLE criteria for kidney failure [5]. Referrals across all medical disciplines, including the intensive care unit (ICU) were included. The definition of AKI was according to the Kidney Disease: Improving Global Outcomes (KDIGO) criteria. In accordance with these guidelines, patients with a rise in serum creatinine of ≥26.5μmol/l within 48 hours, or serum creatinine increase by a multiple of ≥ 1.5 from baseline within the prior 7 days, or fall in urine output of <0.5ml/kg/hour for more than 6 hours, were included. The serum creatinine over the last year was recorded as the baseline. Where baseline serum creatinine was unknown, patients whose serum creatinine improved, independent of dialysis, were assessed as having had an episode of AKI, as described in the KDIGO AKI Guidelines [6]. We elected not to use the Modification of Diet in Renal Disease (MDRD) study equation for estimating baseline serum creatinine as it has been shown to be inaccurate in those with a high baseline serum creatinine [7]. AKI was staged for severity according to the KDIGO criteria. Due to missing patient variables, we were not able to use physiological severity illness scoring methods such as the Acute Physiology and Chronic Health Evaluation (APACHE) score. Instead the AKI severity stage was used as the main marker of patient illness as it has been shown to correlate to mortality outcome [8].

A clerking sheet for AKI patients was designed, and served as a data capture sheet. Patients were contacted at or after 3 months (ninety days) following diagnosis for a repeat serum creatinine. Patients who had abnormal serum creatinine levels were referred for follow up in the Renal Unit.

Patients in whom AKI developed during their hospital admission were labeled as having hospital acquired AKI, and all other patients as community acquired AKI.

In those patients that interchanged dialysis modality, the method at initiation was recorded for purposes of analysis. Time to dialysis initiation from diagnosis of AKI was measured for each patient in days. Dialysis was initiated immediately for refractory hyperkalaemia (K^+^> 6.5 mmol/L), persistent metabolic acidosis pH < 7.2, refractory pulmonary oedema and overt uraemic symptoms. In the absence of the above, timing of dialysis was at the discretion of the nephrologist in consultation with treating specialist.

Outcome measures were mortality at 30 and 90 days, and recovery of renal function at 3 months. Renal recovery was based on the definition of the Acute Dialysis Quality Initiative (ADQI) group, where full renal recovery is return to the baseline Risk Injury Failure End stage renal disease (RIFLE) classification (serum creatinine less than a multiple of 1.5 from baseline), partial recovery if patients persist at a more severe RIFLE class but are not dialysis dependent, and end stage renal disease if they are dialysis dependent after three months [5]. Where the baseline serum creatinine was unknown, patients whose renal function recovered to a level within the laboratory reference range were assessed as fully recovered.

### Statistical analysis

Data was entered into an Excel spreadsheet from the clerking sheet. The data in the Excel spreadsheet was cleaned and imported into Stata 12.1 for analysis (Stata Corporation, College Station, TX, USA). All statistical hypotheses were carried out at a 5% level of significance and 95% confidence intervals (CI) were reported. For categorical data, proportions were described and the chi squared test and its variants was used to test associations.

Continuous data was tested for *normality* using the Shapiro-Wilks test. For *normally* distributed data, means and standard deviations were described while medians and interquartile ranges (IQR) were described for *non-normally* distributed data. The *t-test* and its variants was used to test associations between *normally* distributed variables and groups while the Mann-Whitney U test and its non-parametric variants was used to test associations in *non-normally* distributed data. Odds Ratios (OR), P-values and 95% confidence intervals were used to describe associations. Univariate relationships for potential determinants of mortality at three months were tested and reported. Epidemiological variables, co-morbidities and management variables were considered. Multivariate analysis of determinants of mortality at three months was carried out using forward stepwise logistic regression, with a 0.05 significance level for addition and a 0.15 significance level for removal of variables. We included variables with a P-value of less than 0.20 from the univariate analysis. Demographic variables considered as confounders were age, gender and ethnicity. Further analysis restricted the analysis to dialysed patients only. The effect of the timing of dialysis was explored, with the time (days) to dialysis from AKI onset categorised into early (1 day), delayed (2–5 days) and late (>5 days). This categorisation of timing of dialysis was based on that used in the Beginning and Ending Supportive Therapy for the Kidney (BEST KIDNEY) study (7).

## Results

There were 10,750 hospital admissions over this period and a total of 366 patients was included giving an incidence of 3.4% of admissions. There were 214 (58.5%) males and 152 (41.5%) females. The median age was 44 years (IQR 14–82). Most, 265 (72.4%) had community acquired AKI. More than half were medical referrals, 217 (59.3%), and the remainder predominately from surgery (33.9%) and 7.9% from Obstetrics and Gynecology. The majority 307 (83.9%) had stage 3 AKI, including 72 of the 101 (71.3%) patients with hospital acquired AKI.

Co-morbidities included hypertension in 152 (41.9%) patients, HIV in 75 (20.5%), diabetes mellitus in 65 (17.8%), heart disease in 58 (16.1%), chronic kidney disease in 37 (10.1%), including 10 (2.7%) with HIV associated nephropathy of whom 7 were biopsy proven, and cancer in 16 (4.4%). There were 17 (4.6%) pregnant patients.

Sepsis was the most common precipitating factor for AKI present in 222 (60.7%) of patients. (Table 1). Sepsis was diagnosed if a patient had a definite or suspected infection, with systemic manifestations encompassing the systemic inflammatory response syndrome of either pyrexia or hypothermia, tachypnea, tachycardia or change in leucocyte count. It was a common finding among those with hospital and community acquired AKI, present in 63 out of 101 (62.4%) and 159 out of 265 (60%) respectively.

**Table 1:** Precipitants and causes of AKI.

AKI precipitant or cause	Total (% of 366)
**Sepsis (as either the sole cause or one of the causes)**	**222/366 (60.7%)**
Pneumonia	42(11.5%)
Pyelonephritis/urosepsis	27(7.4%)
Sepsis post trauma	24 (6.6%)
Acute gastrointestinal tract pathology with sepsis	24 (6.6%)
Soft tissue sepsis	6 (1.6%)
Bacterial gastroenteritis	4 (1.1%)
Meningitis	2 (0.55%)
Septic arthritis	2 (0.9%)
Septic abortion (also later in this table)	1 (0.27%)
Other/miscellaneous sites of sepsis	90 (24.6%)
**Toxins**	**137/366 (37.4%)**
IV contrast	50 (13.7%)
Tenofovir	23 (6.3%)
Aminoglycosides	10 (2.7%)
Herbal toxins	7 (1.9%)
*Other exogenous toxins	47 (12.8%)
**Non cardiovascular and non obstetric surgery related**	**66 (18.0%)**
**Gastroenteritis or vomiting or dehydration**	**47 (12.8%)**
**Trauma**	**41 (11.2%)**
**Cardiovascular surgery related**	**30 (8.2%)**
**Obstruction**	**17 (4.6%)**
**Pancreatitis**	**16 (4.4%)**
**Pre-eclampsia**	**14 (3.8%)**
**Obstetric haemorrhage**	**4 (1.1%)**
**Multiple myeloma**	**3 (0.82%)**
**Abdominal compartment syndrome**	**1 (0.27%)**
**Septic abortion**	**1 (0.27%)**
**Thromboembolic (renal infarction)**	**1 (0.27%)**

* Other exogenous toxins

Non-steroidal anti-inflammatory drugs (5), ethylene glycol (4), methamphetamine locally called ‘Tik’ (2), heroine (1), angiotensin converting enzyme inhibitor (1), methanol (1), antipsychotic medication related rhabdomyolysis (1), ethanol related organ dysfunction (7). The remainder were mostly assessed as related to antibiotics including amphotericin B, colistin, cotrimoxazole and rifampicin.

Pre-renal causes of AKI included gastroenteritis and obstetric haemorrhage (Table 2).

**Table 2:** Pathological diagnoses of AKI (including biopsy proven cases).

Variable	Total (% of 366)
ATN predominant (including 11 histological diagnoses)	272 (72.1%)
Glomerulonephritis/vasculitis (including 1 clinical diagnosis)	20 (5.5%)
Pyelonephritis (including 5 histological diagnoses)	19 (5.2%)
Obstruction	17 (4.6%)
Acute interstitial nephritis (including 2 histological diagnoses)	14 (3.8%)
Pre-eclampsia	14 (3.8%)
Granulomas	8 (2.2%)
Malignant hypertension (including 3 histological diagnoses)	7 (1.9%)
Multiple myeloma	3 (0.82%)

Clinically acute tubular necrosis was the most common underlying cause of AKI, in 272 (72.1%) patients. It was due to exogenous nephrotoxins in 137 (37.4%) patients (Table 2).

Among the 75 patients with HIV, there were often multiple potential causes of AKI in a single patient. Some causes were ones that may be expected in HIV such as pneumonia and gastroenteritis, and others were not specific to HIV such as post-partum haemorrhage (S2 Table). Notably 6.3% of patients with HIV had recent use of tenofovir as part of their treatment regimen. In all of these patients, tenofovir was a presumed contributor to AKI. Within the limitations of this study, no objective evidence was sought to substantiate tenofovir as the cause of AKI. We did not look for evidence of tenofovir induced proximal tubular injury such as phosphaturia and glycosuria or renal biopsy ultrastructural findings of giant mitochondria.

A biopsy proven diagnosis of AKI was obtained in 36 (9.8%) patients and 9 had more than one histological diagnosis (S1 Table). The causes of biopsy proven AKI, as a proportion of the 36 patients who were biopsied were glomerulonephritis (52.8%), acute tubular necrosis (30.6%), malignant hypertension (8.3%), pyelonephritis (8.3%), acute interstitial nephritis (5.6%), and granulomas (22.2%) (S1 Table).

Granulomas were detected in 8 patients, 4 of whom were HIV positive. Among the 4 HIV positive patients, opportunistic infection was thought to be likely and arrangements were made for patients to have cultures to test for mycobacterium tuberculosis and fungal infections. One of them had positive Ziehl-Neelsen staining and within the clinical context, mycobacterium tuberculosis was diagnosed. Among the HIV negative patients one was diagnosed with renal mycobacterium tuberculosis. A second HIV negative patient had features of an acute drug reaction. A third HIV negative patient had additional biopsy findings of necrotising vasculitis and clinical features of Henoch-Schonlein Purpura. The fourth HIV negative patient had accompanying mesangial proliferation and acute tubular necrosis. This patient did not return for follow up and further investigations.

Other causes of AKI were urinary tract obstruction, trauma including rhabdomyolysis from mob violence, multiple myeloma, 1 patient with renal infarction, and 1 with abdominal compartment syndrome (Table 1). AKI occurring post cardiovascular surgery was detected in 30 (8.2%) patients and post non-cardiovascular surgery in 66 (18.0%) patients (Table 1).

There were 202 (55.2%) ICU managed patients, while 204 (60.7%) were dialysed. A total of 145 (39.6%) patients had both ICU admission and dialysis. Of the 204 dialysed patients, 5 did not have a clear record of the first dialysis modality. Of the remaining 199 with clear data on first dialysis modality most, 88 (44.2%) were on intermittent haemodialysis, 72 (36.2%) had sustained low efficiency daily dialysis (SLEDD), and 39 (19.6%) had continuous veno-venous haemodialysis (CVVHD). The mean time to dialysis was one day (IQR 1–2, Range 1–21). The 30 day mortality was 31.2% (114 patients) and the 90 day mortality was 38.8% (142 patients). Although an increase in both 30 and 90 day mortality was seen with increasing severity stage of AKI, multivariate analysis did not identify this as an independent predictor of mortality (Tables 3 and 4).

**Table 3:** 30 and 90 day mortality and associated patient characteristics.

	30 day mortality, n (%)				90 day mortality, n (%)			
Department	Medical	Surgical	Obstetrics & Gynaecology	Overall	Medical	Surgical	Obstetrics & Gynaecology	Overall
**Total dialysed patients**	48/120 (40.0)	28/73 (38.4)	1/12 (8.3)	77/205 (37.6)	56/120 (46.7)	37/73 (50.7)	3/12 (25.0)	96/205 (46.8)
**Total ICU patients**	34/89 (38.2)	43/105 (41.0)	1/8 (12.5)	78/202 (38.6)	35/89 (39.3)	54/105 (51.4)	3/8 (37.5)	92/202 (45.5)
**ICU dialysed patients**	30/72 (41.7)	28/66 (42.4)	1/7 (14.3)	59/145 (40.7)	31/72 (43.1)	37/66 (56.1)	3/7 (42.9)	71/145 (49.0)
**Total AKI patients**	67/217 (30.9)	45/124 (36.3)	2/25 (8.0)	114/366 (31.2)	81/217 (37.3)	58/124 (46.8)	3/25 (12.0)	142/366 (38.8)
**Total AKI 1 (n = 27)**				2/27 (7.4)				4/27 (14.8)
**Total AKI 2 (n = 32)**				9/32 (28.1)				12/32 (37.5)
**AKI 3 (n = 307)**				103/307 (33.6)				126/307 (41.0)

**Table 4:** Univariate and multivariate analysis of potential risk factors for 90 day mortality.

Variable	Univariate analysis OR p-value 95%CI	Multivariate analysis OR p-value 95%CI
Age	1.02 0.021 1.00–1.03	0.99 0.615 0.97–1.02
Surgical 58/124 (46.8%)	1.65 0.026 1.06–2.57	1.34 0.348 0.72–2.52
Sepsis 102/222 (45.9%)	2.21 0.001 1.41–3.47	1.83 0.066 1.02–3.27
Hypertension 61/152 (40.1%)	1.10 0.669 0.72–1.68	1.04 0.874 0.64–1.90
HIV 30/75 (40.0%)	1.07 0.801 0.64–1.80	1.11 0.929 0.44–2.12
AKI stage	1.70 0.015 1.11–2.61	1.31 0.276 0.81–2.11
Dialysis 96/204 (47.1%)	2.24 <0.001 1.45–3.47	0.78 0.865 0.04–13.99
ICU 92/202 (47.5%)	1.89 0.004 1.23–2.93	1.27 0.408 0.72–2.24
Dialysis + ICU 74/145 (51.0%)	2.06 0.001 1.33–3.17	1.60 0.212 0.77–3.34
First SLEDD 38/72	2.04 0.007 1.21–3.44	0.80 0.642 0.32–2.02
First CVVHD 23/89	2.51 0.008 1.28–4.94	2.21 0.179 0.70–7.02
First HD 34/88	0.99 0.972 0.61–1.62	0.74 0.593 0.24–2.24
Mechanical ventilation 58/107	2.76 <0.001 1.69–4.51	2.46 0.019 1.41–4.30
Inotropes 43/84	2.27 0.001 1.37–3.76	1.24 0.618 0.53–2.91
Time to dialysis	1.14 0.073 0.99–1.32	1.93 0.080 0.93–4.03
AKI stage 1 versus stages 2 and 3 combined	1.87 0.047 1.03–3.47	1.20 0.637 0.57–2.60

A comparison of AKI stage1 to stages 2 and 3 combined did not affect mortality outcome on multivariate analysis (Table 4). On univariate analysis, the use of both SLEDD and CVVHD were associated with mortality but not on multivariate analysis (Table 4).

The 90 day mortality of patients managed in the ICU was 45.5% (92 of 202) rising to 49.0% (71 of 145) among ICU patients who were dialysed. Mortality rates increased with increasing patient age but no mortality association was detected on multivariate analysis. Multivariate analysis detected mechanical ventilation as the only independent predictor of 90 day mortality (OR 2.46, p = 0.019, 95% CI 1.41–4.30). However, a borderline association with mortality was seen with sepsis (OR 1.83, p = 0.066, 95%CI 1.02–3.27), and prolonged time to dialysis (OR 1.93, p = 0.080, CI 0.93–4.03). Sepsis was a significant finding among those with hospital acquired AKI who were dead at 90 days (p-value 0.005), and the majority of these (39/50, 79.8%) had stage 3 AKI.

A follow up serum creatinine at or after 90 days was only present in 119 (53.1%) of the 224 patients who were alive at 90 days. Of these returning patients, complete renal recovery occurred in 95 (79.8%) and 4 (3.4%) developed end stage renal disease. All of the patients going onto end stage renal disease had an abnormal baseline serum creatinine. Of the 57 returning patients who were managed in ICU but not dialysed, 47 (82.5%) had full renal recovery. Among the 40 returning patients who were admitted to ICU and dialysed, 31 (77.5%) had full renal recovery.

A comparison of patients returning or not returning for 3 month serum creatinine revealed that a greater proportion of the former were mechanically ventilated (p = 0.017) or older (p = 0.049).

## Discussion

The epidemiology of AKI in the study overlapped features described in developing and developed countries. There were young patients, reported to be a predominant proportion of those presenting with AKI in developing countries and elderly patients reported to be more common in developed countries (age range 13–91 years). In addition a notable percentage of patients were both ICU managed and dialysed (51%), in keeping with the experience of urban areas of other developing countries [9]. In keeping with studies of adults worldwide, there was a predominance of male patients [10].

The incidence of AKI in our study, of 3.4%, was very low compared to other studies of AKI in hospitalized patients. A study in the United States of America by Wang et al reported an AKI incidence of 22.7% among 19249 hospitalised patients [11]. In their meta-analysis of AKI studies, Susantitaphong et al reported a pooled world-wide AKI incidence of 21.6% among adults, primarily in hospital settings [12]. The very low figure in our study may reflect low referral rates of patients with early stages of AKI to the Renal Unit.

Risk factors for AKI included non-communicable diseases (such as hypertension, diabetes mellitus, and heart failure), obstetric causes and infectious diseases (HIV). A large proportion of patients (41.5%) had hypertension as a risk factor. Hypertension is a potential cause of chronic kidney disease, a recognized risk factor for AKI. Our finding may be due to the increasing prevalence of this condition in the South African population [13].

Similarly to other developing countries, the majority of patients (72.4%) had community acquired AKI [6]. Although patients with hospital acquired AKI were in the minority (27.6%), the majority of these patients (71.3%) had stage 3 AKI and sepsis (62.4%). This high percentage may be explained by a delay in detection of deterioration in the clinical condition of hospitalized patients, resulting in late referral.

We found that sepsis was the most common cause of AKI across all the referring disciplines (60.7%). Among patients who had community acquired AKI this may be a reflection of the burden of communicable diseases in South Africa. Among those with hospital acquired AKI, this may reflect the rates of nosocomial infections in the hospital, such as Acinetobacter baumanii in the ICU setting [14].

The proportion of patients with urinary tract obstruction (4.6%) is lower than that found in studies conducted in Sudan, where the percentage of patients in whom obstruction was a cause of AKI ranged from 12.3% to 27.9% [15].

A histopathological cause of AKI was obtained in 36 (9.8%) patients who had a renal biopsy, some of whom had more than one histological finding. In many patients, such as those with glomerulonephritis and acute interstitial nephritis, treatment was tailored to the biopsy diagnosis. In keeping with previously published data from the institution on the pattern of glomerulonephritis, mesangiocapillary (membranoproliferative) glomerulonephritis was the most frequently detected primary glomerulonephritis [16].

One of the aims of the study was to identify potential areas of prevention of AKI. In our study, the majority of patients with hospital acquired AKI had stage 3 disease (71.2%), suggesting that the opportunity for earlier intervention to prevent progression from less severe disease was missed. A large proportion of these patients (62.4%) also had sepsis, which was associated with mortality (p-value 0.005). It may be that earlier detection and management of sepsis in these in-hospital patients could have prevented the development of AKI, similarly to findings of a National Confidential Enquiry into Patient Outcome and Death report in the United Kingdom [17].

The mortality rate among ICU patients requiring dialysis (49%) was higher than reports from the multicentre Finnish AKI (FINNAKI) study, of patients in Finnish ICUs, which reported a 90 day mortality rate of 33.7% [18]. The strongest independent predictor of mortality on multivariate analysis in our study was mechanical ventilation (OR 2.46, p-value 0.019, 95% CI 1.41–4.03). This finding was similar to the finding in the multinational and multicentre study of acute renal failure in critically ill patients conducted by Uchino, Kellum et al which found mechanical ventilation to be one of the independent risk factors for in hospital mortality [19]. One explanation for this finding may be the possibility of deleterious cytokine mediated organ cross-talk between the kidneys and distant organs [20]. Additionally, there is evidence, from both animal and human studies, to suggest that primary acute lung injury itself may contribute to AKI, as illustrated by findings from the first Acute Respiratory Distress Syndrome Network Trial conducted from 1996 to 1999. This study found that 24% of patients with acute respiratory distress syndrome developed AKI (where it was defined as a serum creatinine level higher than 177μmol/l) within the first four days of enrolment in the study [21]. Variables carrying a borderline significant association with mortality on multivariate analysis in our study were sepsis (OR 1.83, p-value 0.066, 95% CI 1.02–3.27), and time to dialysis (OR 1.93, p-value 0.080, 95% CI 0.93–4.03). This was consistent with previous studies that identified both as carrying mortality associations [7,22]. Although our findings demonstrated an increase in 30 and 90 day mortality rates with increasing severity stage of AKI, it was not an independent predictor of 90 day mortality on univariate analysis. The large proportion of patients with stage 3 AKI may have been a confounder in the ability to detect the effect of the stage of AKI on mortality in our patients.

The percentage of patients with partial renal recovery (16.8%) is a cause for concern in a country with limited access to renal replacement therapy. Such patients are at risk of deteriorating to end stage renal disease and require long term follow up [7,23]. Although comprising a small number, it is a significant finding that all of the patients going onto end stage renal disease had an abnormal baseline serum creatinine. This finding of the acceleration of underlying chronic kidney disease to end stage renal disease after an episode of AKI is consistent with findings in other studies [24,25].

As only 53.1% of the patients who were alive at 90 days were available for a follow up serum creatinine, we compared the characteristics of the patients with and without a follow up serum creatinine to detect differences between them that could affect our interpretation of the pattern of renal recovery in our study population. We found more mechanically ventilated patients among those returning (p-value 0.017), suggesting that they were more ill. We also found that these patients were slightly older (p-value 0.049). Other comparisons incorporating data on demographics, management and sepsis found no differences between the two groups.

Although the study was not adequately powered to make definitive conclusions about the effect of timing of dialysis on patient outcome, there was a borderline increase in mortality risk with delayed chronological time to dialysis (OR 1.93, p-value 0.080, 95% CI 0.93–4.03). This was in keeping with findings in the BEST KIDNEY study, where delayed chronological time to dialysis was found to be a better mortality indicator than serum urea and creatinine levels, likely related to the potential confounders with the use of these biomarkers [7].

An important limitation of the study was the low incidence of AKI stages 1 and 2. The reason is that dialysis services are not available at our secondary and peripheral hospitals, and Groote Schuur Hospital is the major regional referral centre. Patients referred to our centre for dialysis are more likely to have AKI stage 3. We have no data on the incidence of AKI 1 and 2 in these referral hospitals.

In conclusion, this is the first study to look at the epidemiology, management and 3 month outcome of the full spectrum of AKI among patients referred to the Groote Schuur Hospital Renal Unit. We found that AKI carries a high mortality risk and that this risk is highest among mechanically ventilated patients. We also found that sepsis and delayed time to dialysis may be associated with a high mortality risk.

A significant proportion of patients with hospital acquired AKI had stage 3 AKI and sepsis. Based on this finding, we recommend that efforts be made to enable early detection and treatment of deteriorating patients, such as an early warning score, as used in the United Kingdom [26].

We found that a significant number of patients did not have full renal recovery, a factor that will contribute to the burden of chronic kidney disease in the region. This reinforces the importance of a management plan that requires patient follow up at 3 months following an episode of AKI.

Lastly, we found that while HIV is an important co-morbidity to consider in South Africa, it was not a predictor of mortality in the patients that we studied.

## Supporting information

S1 Table Renal biopsy results. (PDF)

S2 Table Causes of AKI amongst HIV positive patients. (DOCX)

S3 Table Excel spreadsheet of study datasets. (XLSX)
