# Supplementary material for: A prospective study of the demographics, management and outcome of patients with acute kidney injury in Cape Town, South Africa
Source: PLoS One. 2017 Jun 1;12(6):e0177460. doi: 10.1371/journal.pone.0177460 (PMC5453519; doi:10.1371/journal.pone.0177460)
Supplement: S1 Table — (PDF) [file pone.0177460.s001.pdf]

# S1 Table – renal biopsy results (total = 36 patients)

| PATIENT | RENAL BIOPSY RESULT |
|---|---|
| 1 | Acute tubular necrosis, granulomas – Ziehl-Neelsen stain positive |
| 2 | HIV associated nephropathy, granulomas |
| 3 | Ascending pyelonephritis |
| 4 | Crescents, mesangiocapillary (membranoproliferative) glomerulonephritis |
| 5 | Acute tubular necrosis, granulomas |
| 6 | Lupus nephritis class V, necrotising lesions, crescents |
| 7 | Acute post infectious glomerulonephritis |
| 8 | Necrotising lesions, granulomas |
| 9 | Acute tubular necrosis, interstitial HIV associated nephropathy |
| 10 | Mesangiocapillary glomerulonephritis, acute tubular necrosis, granulomas |
| 11 | HIV associated nephropathy, acute interstitial nephritis |
| 12 | Mesangiocapillary glomerulonephritis, acute tubular necrosis |
| 13 | Mesangiocapillary glomerulonephritis, crescents |
| 14 | Mesangiocapillary glomerulonephritis, crescents, lupus nephritis class IV |
| 15 | Mesangiocapillary glomerulonephritis, crescents, ? lupus nephritis class IV, acute tubular necrosis |
| 16 | Mesangiocapillary glomerulonephritis, ? lupus nephritis class IV |
| 17 | Acute tubular necrosis |
| 18 | Necrotising lesions, IgA |
| 19 | Acute post streptococcal glomerulonephritis/post infectious glomerulonephritis |
| 20 | Acute mesangiocapillary glomerulonephritis, crescents, acute interstitial nephritis |
| 21 | Acute post streptococcal glomerulonephritis/post infectious glomerulonephritis |
| 22 | Eosinophils, granulomas |
| 23 | HIV associated nephropathy |
| 24 | Acute tubular necrosis, HIV associated nephropathy |
| 25 | Granulomas, acute tubular necrosis, interstitial fibrosis |
| 26 | Hypertension with crenation,? acute tubular necrosis, interstitial fibrosis |
| 27 | Acute tubular necrosis – exclude light chain deposition disease |
| 28 | Acute post streptococcal glomerulonephritis |
| 29 | HIV associated nephropathy, malignant hypertension, ascending pyelonephritis |
| 30 | Malignant hypertension, ascending pyelonephritis, interstitial fibrosis |
| 31 | Membranous glomerulonephritis, HIV associated nephropathy – foetal variant, hypertension |
| 32 | Malignant hypertension |
| 33 | Mesangiocapillary glomerulonephritis, crescents, lupus nephritis class IV |
| 34 | Acute post infectious glomerulonephritis, crescents |
| 35 | Mesangiocapillary glomerulonephritis |
| 36 | HIV associated nephropathy, granulomas, ascending pyelonephritis |
