# Supplementary material for: A prospective study of the demographics, management and outcome of patients with acute kidney injury in Cape Town, South Africa
Source: PLoS One. 2017 Jun 1;12(6):e0177460. doi: 10.1371/journal.pone.0177460 (PMC5453519; doi:10.1371/journal.pone.0177460)
Supplement: S2 Table — (DOCX) [file pone.0177460.s002.docx]

**S2 Table - causes of AKI amongst HIV positive patients**

| **Patient age (years), sex (female,F or male,M)** | **AKI associations per patient** | **Renal biopsy results** |
| --- | --- | --- |
| 1. 27, M | Gastroenteritis |  |
| 1. 51, M | Gastroenteritis and tenofovir |  |
| 1. 37, M | Sepsis, transaminitis - hepatitis, pancytopaenia |  |
| 1. 29, M | Epididymorchitis, dehydration | Acute tubular necrosis, Ziehl-Neelsen stain positive granulomas |
| 1. 72, F | Sepsis |  |
| 1. 25, F | Pneumonia |  |
| 1. 31, M | Tuberculosis, granulomas | HIV associated nephropathy, granulomas |
| 1. 31, F | Sepsis | Ascending pyelonephritis |
| 1. 44, M | Sepsis, tuberculosis, upper gastrointestinal bleed |  |
| 1. 34, F | Gastroenteritis, ?meningitis, tenofovir |  |
| 1. 40, F | Gastroenteritis, sepsis, tenofovir |  |
| 1. 25, F | Pneumonia |  |
| 1. 24, M | Ileus, disseminated tuberculosis, cryptococcal meningitis, amikacin, tenofovir |  |
| 1. 44, F | Pneumonia, tuberculosis, herbal medication, vomiting |  |
| 1. 37, F | Pre-eclampsia, intrauterine death |  |
| 1. 31, M | Gastroenteritis, tenofovir |  |
| 1. 39, M | ?Tenofovir, acute tubular necrosis, granulomas | Acute tubular necrosis, granulomas |
| 1. 38, F | Pyrexia of unknown origin, liver dysfunction - hepatitis, tenofovir |  |
| 1. 60, M | Pneumonia, diarrhea, tenofovir |  |
| 1. 63, F | Sepsis |  |
| 1. 38, F | Pneumonia |  |
| 1. 39, M | Motor vehicle accident – cervical and thoracic spine injury, nosocomial sepsis |  |
| 1. 37, M | Pneumonia, tuberculosis |  |
| 1. 54, M | Pneumonia, tuberculosis, diarrhoea |  |
| 1. 32, M | Tuberculosis, diarrhoea, sepsis, acute hepatitis B, amikacin, spironolactone |  |
| 1. 40, M | Pneumonia +tuberculosis |  |
| 1. 32, M | Pneumonia, mesangiocapillary (membranoproliferative) glomerulonephritis |  |
| 1. 44, F | Septic shock, gastroenteritis |  |
| 1. 44, M | Tenofovir, sepsis |  |
| 1. 38, F | Abruptio placentae – haemorrhage |  |
| 1. 36, M | Tenofovir, rifampicin | Acute tubular necrosis, interstitial HIV associated nephropathy |
| 1. 29, F | Abruptio placentae, post-partum haemorrhage, tenofovir |  |
| 1. 36, M | Non-steroidal anti-inflammatory drugs, tuberculosis |  |
| 1. 40, F | Diarrhoea, seizures, pulmonary tuberculosis, sepsis | Interstitial nephritis and HIV associated nephropathy |
| 1. 26, F | Vomiting |  |
| 1. 75, M | Vomiting – dehydration, drug induced liver injury, tuberculosis, sepsis, tenofovir |  |
| 1. 31, F | Necrotizing glomerulonephritis/Henoch Schonlein purpura, sepsis | Necrotizing lesions, IgA staining |
| 1. 28, M | Tik (methamphetamine substance use) – rhabdomyolysis, cardiogenic shock |  |
| 1. 50, M | Pneumonia, acute liver injury |  |
| 1. 42, M | Syphilitic aortic regurgitation, sepsis |  |
| 1. 40, M | Vomiting, rifampicin, tenofovir |  |
| 1. 63, M | Post abdominal aortic aneurysm repair, sepsis |  |
| 1. 29, M | Pneumonia, tuberculosis on treatment | Acute post streptococcal glomerulonephritis |
| 1. 28, M | ?Tenofovir related acute tubular necrosis or rifampicin and Bactrim (cotrimoxazole) related acute interstitial nephritis |  |
| 1. 53, M | Upper gastrointestinal bleed, amphotericin B, pneumonia |  |
| 1. 35, F | Vomiting – dehydration, shock, pyelonephritis | HIV associated nephropathy |
| 1. 44, F | Tenfovir, rifampicin |  |
| 1. 55, F | Pneumonia, tenofovir |  |
| 1. 37, F | Sepsis, miscarriage |  |
| 1. 38, F | Acute leukaemia |  |
| 1. 48, M | Pyuria –frank pus |  |
| 1. 37, M | Tenofovir, rifampicin | Acute tubular necrosis, HIV associated nephropathy |
| 1. 29, F | Cariogenic shock from supraventricular tachycardia, sepsis, lactic acidosis secondary to zidovudine |  |
| 1. 29, M | Diarrhoea, new HIV diagnosis |  |
| 1. 62, M | Pneumonia |  |
| 1. 64, F | Pneumonia, vomiting, diarrhoea |  |
| 1. 36, F | Malignant hypertension, tenofovir | Malignant hypertension, ascending pyelonephritis, HIV associated nephropathy |
| 1. 50, M | Urosepsis, tenofovir |  |
| 1. 30, M | Pneumonia , tenofovir, Bactrim (cotrimoxazole) |  |
| 1. 42, M | Septic shock – pneumonia, pancreatitis |  |
| 1. 41, F | Tenofovir |  |
| 1. 37, F | Urosepsis, tubo-ovarian abscess, obstructive uropathy |  |
| 1. 44, F | Salmonella non-typhi sepsis | Membranous glomerulonephritis, HIV associated nephropathy (foetal variant) |
| 1. 42, M | Diarrhoea, sepsis |  |
| 1. 55, M | Neutropaenic sepsis,? intravenous contrast nephropathy and ? chemotherapy |  |
| 1. 38, M | Tenofovir, Bactrim (cotrimoxazole), rifampicin, Multi Drug Resistant tuberculosis |  |
| 1. 30, F | Diarrhoea, vomiting, cryptococcal meningitis, candidiasis, sepsis, tenofovir |  |
| 1. 37, M | ?Tenofovir |  |
| 1. 36, F | Incomplete abortion, sepsis, Disseminated intravascular Coagulopathy |  |
| 1. 26, F | Sepsis, Stevens Johnson syndrome secondary to Bactrim (cotrimoxazole) |  |
| 1. 32, F | Multi Drug Resistant tuberculosis, lymphoma |  |
| 1. 31, F | Ascending pyelonephritis |  |
| 1. 44, M | Ascending pyelonephritis, granulomas | HIV associated nephropathy, granulomas, ascending pyelonephritis |
| 1. 41, M | Pancreatitis, sepsis |  |
| 1. 31 ,F | Septic abortion |  |
